# Effect of static pressure on ultrasonic liquid phase exfoliation of few-layer graphene

**DOI:** 10.1016/j.ultsonch.2024.106863

**Published:** 2024-03-28

**Authors:** Hu Zong, Minhui Gao, Aziz Ul Hassan Mohsan, Yibiao Lin, Ying Zhou, Lei Yu, Su Zhao, Yifei Li, Jiahao Zhang

**Affiliations:** a College of Chemical Engineering, Zhejiang University of Technology, Hangzhou 310014, China; b Ningbo Institute of Materials Technology and Engineering, Chinese Academy of Sciences, Ningbo 315201, China

**Keywords:** Static pressures, Ultrasonic liquid phase exfoliation, Graphene, Large-scale production

## Abstract

Ultrasonic Liquid Phase Exfoliation (LPE) has gathered attention from both scientific and industrial communities for its accessibility and cost-effectiveness in producing graphene. However, this technique has faced challenges such as low yield and long production time. In this study, we developed a cyclic ultrasonication system to exfoliate expanded graphite (EG) by applying static pressure to a flow chamber to address these challenges. Using deionized water (DIW) as solvent and polyvinylpyrrolidone (PVP) as dispersion, we obtained graphene slurries with an average lateral size of 7 μm and averaged number of layers of 3.5 layers, after 40 min of ultrasonication. After centrifugation, the yield of single and bilayer graphene was approximately 16 %. The findings showed that regulating hydrostatic pressure can effectively affect the lateral size and number of layers of few-layer graphene. The proposed method is of good potential for scaled-up production of few-layer graphene.

## 1 Introduction

Traditional methods for large-scale graphene production mainly include the Hummer method and chemical vapor deposition (CVD) [1], [2], [3], [4]. These approaches have been crucial in developing graphene, but they have some limits. For instance, graphene produced via the Hummers method often bears defects that can detract from its physical and chemical properties. CVD can yield high-quality graphene. But it relies on metal catalysts and high-temperature processes, which hinders its mass production capacity. Unlike the methods above, ultrasonic LPE is based on physical separation of graphene from inexpensive graphite material utilizing ultrasonic cavitation effect. This method preserves the exceptional characteristics of graphene and reduces the production costs [5]. Consequently, LPE has unlocked considerable potential for applications that demand superior electrical and thermal conductivities, such as in battery electrode materials and thermal interface materials for heat dissipation [6], [7].

The systems can be classified into three primary types: organic solvents, ionic liquids, and aqueous solutions. Organic solvents, such as N-methyl-2-pyrrolidone (NMP), exhibit a surface tension of 40 mN/m, approaching the surface energy of graphene (46.7 mN/m), which helps to weaken the van der Waals forces between graphite layers and enables the quick preparation of high-quality graphene dispersions [8]. However, the toxicity of these solvents poses safety concerns. Ionic liquids are liquid salts with surface energies comparable to graphene [9], [10]. Although they have the potential to stabilize dispersions effectively, their preparation process is relatively complex and expensive. On the other hand, water is an ideal solvent due to its non-toxicity, low cost, and environmental friendliness. It is a viable solvent for the sustainable, large-scale processing of graphene, particularly when combined with effective dispersing agents [11], [12]. However, obtaining high concentrations of graphene in water remains challenging compared to traditional solvents. Technology advancements in power ultrasound such as the introduction of dual-frequency help to improve the efficiency of graphene exfoliation in water [13]. To achieve maximum dispersion in water, it is essential to optimize the process parameters such as temperature [14], frequency [15], power [5], and processing time [16], [17]. Even the sonotrode tip design can impact the dispersion quality [18]. All these factors are essential in tuning the process to achieve graphene sheets with desired 2D size and number of layers.

Previous research on ultrasonic LPE mainly focused on the connection between cavitation bubbles and the size and thickness of 2D graphene sheets. It has been shown that high-frequency ultrasound (over 100 kHz) produces smaller bubbles with higher cavitation intensity [19], which is beneficial for effective exfoliation of graphene sheets. However, due to design limitations, high frequency ultrasonication devices are usually of low output power and low efficiency. This is the reason why most commercialized high power ultrasonication devices are designed to work at low ultrasonic frequency range from 20 kHz to 40 kHz. On the other hand, correlation between cavitation intensity and static pressure has been extensively investigated in the literature [20], [21], [22], [23], [24], [25], [26]. Raising the static pressure of the liquid media also results in smaller bubbles with higher cavitation intensity. Therefore, this work is focused on optimizing the LPE process by utilizing low frequency ultrasonication under different static pressures. The aim is to enhance both yield and quality of the graphene produced by ultrasonic LPE.

## 2 Material and methods

### 2.1 Materials

Expanded graphite (YH-200) from Qingdao Yanhai Carbon Materials Co., Ltd., was used in the following experiments. The initial particle size of the expanded graphite was 200 mesh (∼74 μm). Deionized water (DIW) and polyvinylpyrrolidone (PVP) from China National Pharmaceutical Group Chemical Reagent Co., Ltd. were selected.

### 2.2 Preparation

**Fig. 1:**
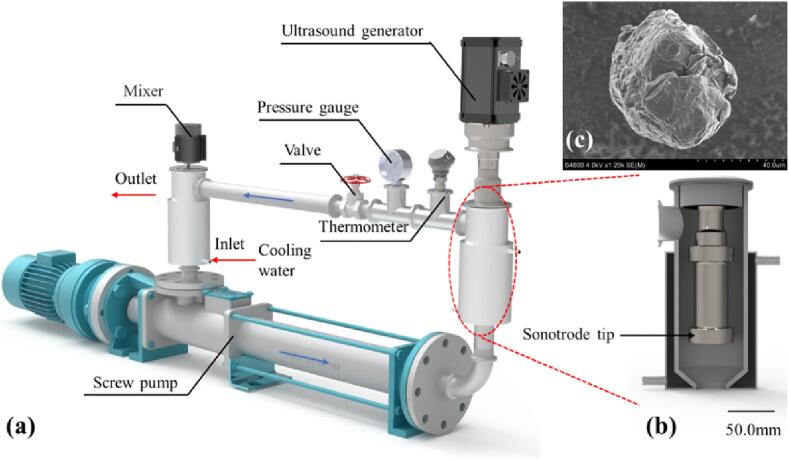
**Schematic drawing of Ultrasonic liquid phase exfoliation apparatus.** (a) The overall design. (b) Sonotrode tip and cavitation chamber. (c) SEM image of expanded graphite raw material.

The schematic drawing of the ultrasonic LPE apparatus is shown in Fig. 1. The slurry is circulated in the pipeline driven by a screw pump. A needle valve is installed to restrict the flow rate by varying its opening. For a fixed pump rotation speed, reducing the valve opening will cause static pressure inside the ultrasonic chamber to rise. To offset flow velocity losses, the speed of the screw pump is adjusted to maintain the flow rate at 3.4 L/min. The flow chamber has a water-cooling jacket to maintain a stable temperature during the experiment. The ultrasonic system is ResoLabD-1500, manufactured by ResoTek (Ningbo) Co., Ltd. The effective power output power is maintained at 1,400 W under different static pressures. This is achieved through regulation the vibration amplitude at the tip of the sonotrode. The actual output power is monitored using an oscilloscope, which calculates the real-time power as: $P=V_{\text{rms}}\times I_{\text{rms}}\times cos\left(\phi \right)$.

For each experiment a mixture weighing 1500.00 g, containing 45.00 g of expanded graphite, 2.25 g of polyvinylpyrrolidone (PVP), and 1452.75 g of deionized water (DIW) was prepared. Following 30 min of stirring, the mixture was transferred into an ultrasonic flow chamber. The temperature of the mixture was maintained at around 40 ℃ by the water jacket to ensure optimum efficiency [14]. Samples were collected at 10-minute interval for subsequent analysis.

### 2.3 Characterization methods

BT-9300 s laser diffraction particle size analyzer was utilized to measure the dimensions of graphene [27]. Scanning electron microscope (SEM) is commonly used for raw material characterization or size measurement. However, graphene agglomeration frequently results in inaccurate size measurements. By implementing vacuum freeze-drying technology, the unique microscopic morphology of the graphene samples was preserved. This process ensured that the samples remained dispersed, making them suitable for further analysis [28], [29]. To observe the morphology of the freeze-dried powder samples, they were evenly distributed onto a conductive adhesive tape and inspected using SEM (Hitachi S-4800) with an accelerating voltage of 4.0 kV.

Raman spectroscopy is an efficient method to evaluate physical characteristics of graphene [30], [31]. Renishaw Raman spectroscopy system with 532 nm wavelength, was utilized to examine the structure of graphene. The sample was positioned on a silicon wafer, and 20–30 stacked samples were chosen randomly for Raman spectra assessment. Spectral data was collected between the range of 1000 cm^−1^ to 3000 cm^−1^.

The dimensions of the graphene sheets were analyzed through a combination of transmission electron microscopy (TEM) and atomic force microscopy (AFM). HRTEM JEOL 2100, operating at an accelerating voltage of 200.0 kV, was employed to capture TEM images. The AFM images were taken using CSPM5500 under tapping mode, and the images were taken in the retrace direction at a scan rate of 1.5 Hz with 256 samples/line. To facilitate the analysis, microgrid and ultrathin microgrid films were utilized as substrates for TEM, while mica sheets served as the sample substrate for AFM. 30 representative samples were selected for statistical analysis under different pressure conditions and time points. The TEM images were processed using *Image J* software to quantify the number of layers in each sheet. Nano-scope Analysis 2.0 was used to process AFM images (with 100 measurement points) and estimate the average thickness. Statistical analysis was conducted to ensure the credibility of the results.

A 4:1 mass ratio of carboxymethyl cellulose (CMC) and graphene slurry was stirred with a magnetic stirrer at 800 rpm and 40 °C for 2.5 h. The resulting mixture was then applied to a substrate using a film applicator. After surface moisture was removed in a temperature-controlled drying oven, the film's thickness and resistance were measured with a thickness gauge and a four-point probe resistivity meter, respectively. The film's conductivity was calculated using the Van Der Pauw (VDP) method [32].

## 3 Characterization of the few-layer graphene

### 3.1 Particle size distribution

**Fig. 2:**
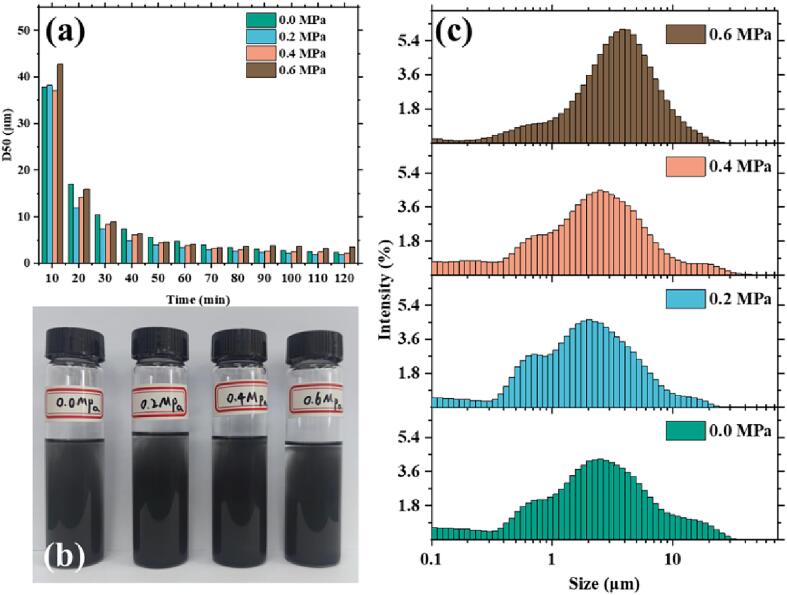
**Tracking of graphene sheets size.** (a) Graphene sheets size (D50 value) reduction over processing time. (b) Final samples (120 min) at different pressures. (c) histograms of particle size distributions.

According to the data presented in Fig. 2a, the D50 values of graphene sheets vary depending on the static pressure applied. The size distribution indicates that the particle size reduction rate for graphene sheets is as following: 0.2 MPa > 0.4 MPa > 0.0 MPa > 0.6 MPa. Size reduction becomes insignificant after 70 min of treatment. However, the treatment was continued for an additional 50 min to find out if the exfoliation continued to occur. Fig. 2b shows the particle size distribution histogram of the diluted (5000 times) sample from 120 min of ultrasonication, which was analyzed using a laser particle size analyzer, as shown in Fig. 2c. Finally, we measured the graphene sheets size (D50) under the four pressure conditions (0.0 MPa, 0.2 MPa, 0.4 MPa, and 0.6 MPa) as 2.3 μm, 1.9 μm, 2.23 μm, and 3.44 μm.

The lateral size of graphene sheets is closely linked to their electrical and thermal properties. Research has shown that as the size of graphene sheets decreases from 200 nm to 1 μm, its thermal conductivity deteriorates up to 300 times and its electrical conductivity deteriorates by a factor of around 10 [33]. The data in Table 1 reveals some interesting findings regarding the impact of pressure on the concentration degree of graphene lateral size. Notably, the results of 0.6 MPa stands out as unique, producing a peak intensity of 6.01 %, which is higher than the other pressure parameters. Additionally, the corresponding span is 50 % smaller compared to the span under 0 Mpa pressure. These results suggest that a pressure of 0.6 MPa enhances the concentration degree of graphene sheets size. However, it is important to note that higher pressure does not always lead to an improved concentration degree of graphene sheets size, as the results for 0.4 MPa are similar to those of 0.0 MPa. In contrast, the results for 0.2 MPa display lower peaks and spans than those of 0.0 MPa and 0.4 MPa.

**Table 1:** **Laser particle size analysis of the final sample**. The results were obtained by analyzing the sample of Fig. 2(b). The lateral size span ($span=\frac{D_{90}-D_{10}}{D_{50}}$) was calculated in four different static pressure conditions.

Static pressure	Peak	Proportion	Span
0 MPa	2.513 μm	4.27 %	4.25
0.2 MPa	2.035 μm	4.66 %	3.16
0.4 MPa	2.613 μm	4.47 %	3.62
0.6 MPa	3.835 μm	6.01 %	2.11

The measurement results suggest that when exposed to ultrasonication under a static pressure of 0.2 MPa, graphene sheet size reduction is the quickest. In contrast, treatment under 0.6 MPa static pressure appears to be gentler, resulting in a more concentrated lateral size distribution. It is worth noting that lateral size stabilizes after 40 min of ultrasonication under 0.6 MPa static pressure, unlike other parameters where size continues to decrease up to 70 min. When evaluating graphene, it is essential to consider microscopic features such as number of layers, thickness, and defects [34], [35], [36]. Further investigation is necessary to explore the mechanism of ultrasonication under different pressures.

### 3.2 SEM & Raman

**Fig. 3:**
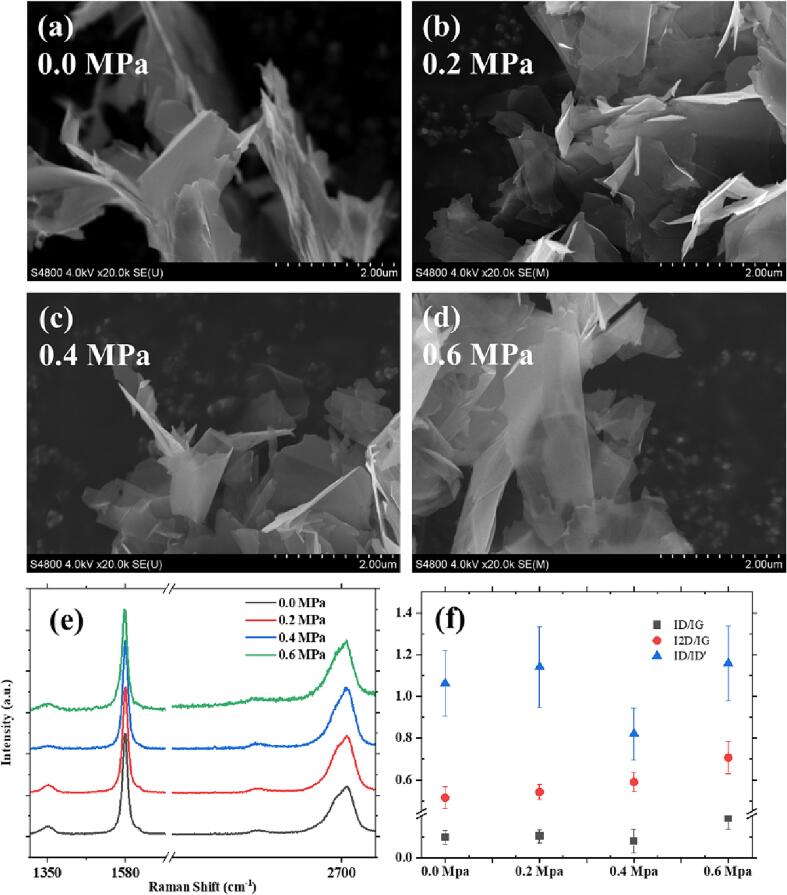
(a) to (d) SEM images of the final (120 min) samples at different pressures. (e)The most representative Raman sample's initial data was chosen for plotting purposes, with a Y-axis shift applied to enhance observation and produce the final figure. (f) Averaged intensity ratios of Raman peaks (D peak, G peak, D' peak, 2D peak).

Fig. 3a to d shows SEM images of graphene sheets structures observed at 20,000x magnification. The transparency of the graphene in the four provided images differs, with (d) > (c) > (b) > (a), indicating that as the pressure increases, the number of graphene layers and their thickness decrease. Since the photographed graphene layers are three-dimensional structures, there may be significant measurement errors in determining their lateral dimension. The exact lateral dimension is measured by the laser diffraction particle size analyzer and TEM data. However, a rough observation suggests that the size is approximately (d) > (a) > (b) ≈ (c), which is consistent with the results obtained from laser particle size analysis.

The sizeable scattering cross section of graphite materials makes Raman spectroscopy an ideal method for analyzing such substances. Individually scanning each graphene sheets can be a tedious and ineffective process since the samples may not be representative. Consequently, the outcome of the experiment was kept as the final sample without undergoing centrifugation to meet the demands of large-scale production. By scanning stacked graphene directly, the average peak intensity can be obtained more promptly, providing a better representation of the average quality of graphene [30], [31], [37], [38]. In the captured Raman spectrum, in addition to the classical characteristic peaks, such as the G peak at 1580 cm^−1^ and the 2D peak at around 2700 cm^−1^, a D peak appeared at 1350 cm^−1^, and a D' peak appeared at 1620 cm^−1^. I_D_/I_G_ can be used as an estimation standard for defect density in graphene. The lowest defect density occurred at a static pressure of 0.4 MPa, with an I_D_/I_G_ ratio of 0.079. In comparison, the values measured under 0.0 MPa and 0.2 MPa were 0.097 and 0.103, respectively. However, it is worth noting that at a static pressure of 0.6 MPa, the I_D_/I_G_ value reached 0.189. This anomaly is generalized because it is a Raman result of a large area scan, and based on the available references, we hypothesize that it appears as a graphene nanosheets outside the range of the laser diffraction particle size analyser [39]. The distance between defects should be < 25 nm [40]. Research has shown that the intensity of the G peak in graphene increases within ten layers [41]. With increasing pressure, the intensity of the 2D peak gradually strengthens, and the I_2D_/I_G_ ratio increases from 0.5 at atmospheric pressure to 0.68. The shape of the 2D peak also becomes narrower. These observations indicate a reduction in the average number of layers in graphene, but it remains as few-layer graphene. The presence of the D peak at 1350 cm^−1^ and the D' peak at 1620 cm^−1^ suggests the existence of certain defects in the obtained graphene, and the defects in graphene increase with increasing pressure. The I_D_/I_D’_ ratios corresponding to sp^3^ hybridization, vacancies, and edge defects are 13, 7, and 3.5, respectively [42], [43]. Regarding the highest pressure, the I_D_/I_D'_ ratio is 1.18, smaller than 3.5, and the edge defects are the predominant type of defect in the graphene sample.

It has been observed that low pressure results in smaller dimensions of graphene in the two-dimensional aspect. And high pressure is helpful to reduce the number of layers and thickness.

### 3.3 TEM & AFM

TEM and AFM are commonly used to observe and describe the number of layers and thickness of graphene. While TEM is useful for observing the number of layers in limited samples, AFM's high-resolution scanning capability enables statistical analysis on a larger scale. Combination the two methods allows a more objective evaluation of graphene's number of layers.

**Fig. 4:**
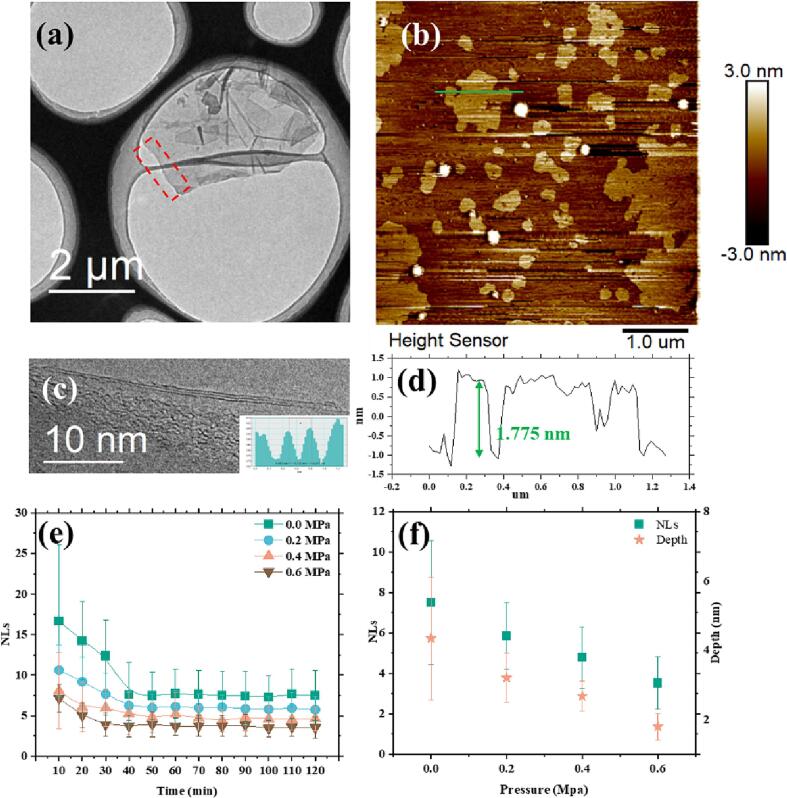
(a) TEM image of final (120 min) sample at 0.6 MPa with (c) higher resolution detail; (b)AFM image of final sample at 0.6 MPa with (d) thickness measurement. (e) The number of graphene layers at different time; (f) The final (120 min) products' relationship between the average AFM thickness values (represented by solid pentagrams) and the average TEM layer values (represented by solid squares).

TEM and AFM images of the sample collected at 0.6 MPa are presented in Fig. 4a-d. According to Fig. 4c, a layer spacing of 0.331 nm was calculated, which approximate to the measurement of 3.44 Å previously reported in Ref. [44]. The height, as determined by AFM, is 1.755 nm (Fig. 4d). Considering the impact of the mica sheets substrate, the bottommost graphene layer is approximately 1 nm [45], [46], and the calculated height of the single-layer graphene is about 0.39 nm. The discrepancy between the results obtained by TEM statistics is that there are wrinkles on the graphene surface. Further statistical analysis of layer at varying pressures and time intervals (as indicated in Fig. 4e) reveals that ultrasonic exfoliation of graphene is more effective with increased static pressure. At standard atmospheric pressure (0 MPa), the mean number of layers is 7.5 with larger variation. However, the introduction of static pressure leads to a substantial reduction in the number of layers with smaller variation. After subjecting the samples to 40 min of ultrasonication, the number of layers reaches to steady state for both standard and pressured conditions. Combined with previous results on graphene lateral size, it can be concluded that exfoliation is completed in the first 40 min while size reduction is completed in 70 min. Fig. 4f shows a linear relationship between number of layers and thickness, which is consistent with the TEM and AFM results.

By combining these data with previously measured lateral dimensions, it can be concluded that ultrasonication under a static pressure of 0.6 MPa for 40 min are the optimal process parameters for graphene with least number of layer (3.5Ls) and largest lateral size (7 μm). The premise of the argument is that all tests were conducted with ultrasonic power of 1400 W applied on 1400 g of slurry. A higher ultrasonic power could further reduce the processing time.

### 3.4 The yield of graphene

Yield is a crucial consideration for large-scale production of graphene. It is important to note that the graphene used in this experiment did not undergo centrifugation, yet still achieved an average layer count of 3.5. Furthermore, the obtained sample requires no additional treatment, making it highly suitable for large-scale production. The solid composition of the sample decreased slightly from 3.00 wt% to 2.89 wt%, resulting in a high yield of 96.30 %.

**Fig. 5:**
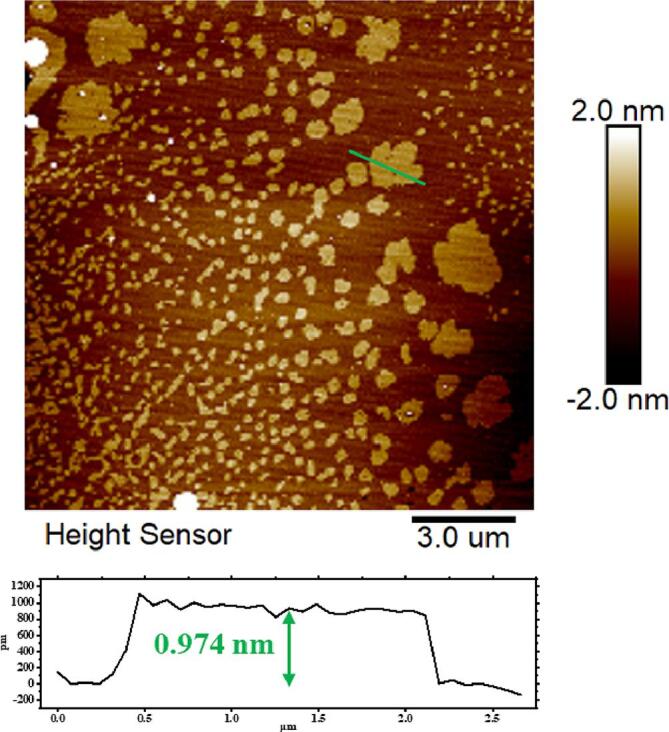
The AFM image of the graphene sheets at 120 rcf for 30 min under optimal process parameters.

Under 0.6 MPa pressure (as depicted in Fig. 5), there is a wide distribution of graphene thickness, with some as thin as approximately 1 nm. This further indicates the presence of bilayer or even single-layer graphene in the samples [2], [34], [47], [48]. To quantify the yield of single-layer graphene and bilayer graphene the samples were send through centrifugation. After centrifugation at a speed of 120 rcf for a duration of 30 min, AFM analysis revealed that the samples had a mean thickness of less than 1.2 nm. By referring to Fig. 4f and analyzing the correlation between the number of layers and the thickness, it is apparent that the sample consists of both single-layer and bilayer graphene. As a result, the yield of single-layer graphene and bilayer graphene in the slurry was 16 %, close to the a maximum of 18 % found in reference [49].

## 4 Electrical conductivity of graphene slurry

CMC, a cellulose derivative, is a linear polymer that produces a fibrous powder with a white or slightly yellow hue. It is entirely safe and odorless, and it dissolves effortlessly in hot and cold water and polar solvents to create clear, thick solutions. These distinctive characteristics make it a desirable binding agent for graphene slurries [50], [51], [52].

**Fig. 6:**
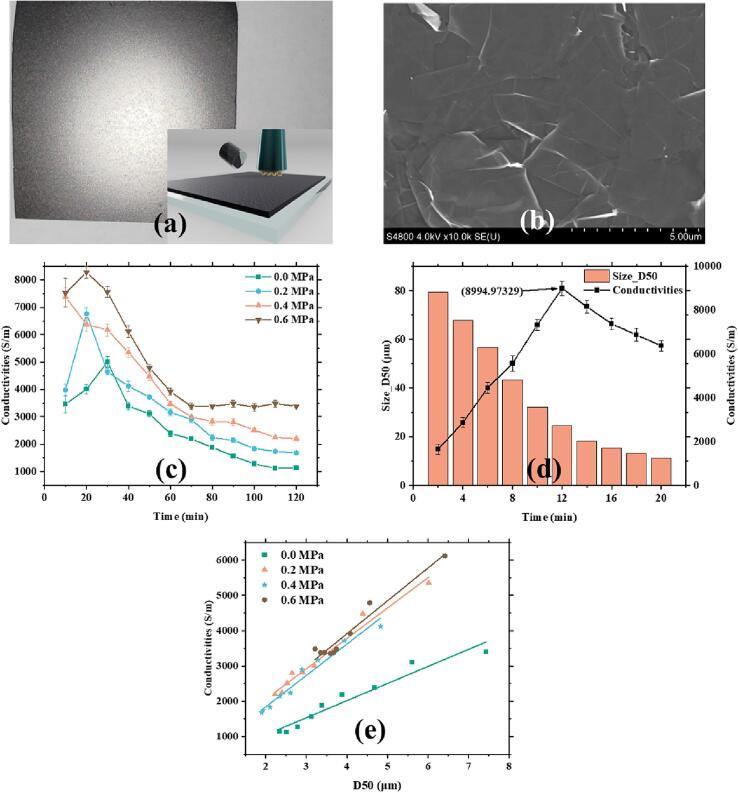
**Electrical conductivity test.** (a) Sample imaging and conductivity testing method. (b) SEM image of PET-based graphene film. (c) Relationship between graphene conductivity and processing time under different static pressures. Additionally, (d) the graph depicts the conductivity changes within the first 20 min under 0.4 MPa static pressure. (e)Normalize the number of graphene layers for each group with ultrasonic processing time ranging from 40 to 120 min to a consistent value and plot a linear relationship graph between D50 and conductivity.

Fig. 6a depicts the PET-based graphene film prepared with CMC as binder. The slurry was mixed thoroughly and then coated onto a smooth PET film. Fig. 6b shows an SEM image of the PET-graphene film. The VDP method was used to measure the material's conductivity. Fig. 6c shows time-dependent variation in the conductivity of the graphene film under different static pressure conditions. Overall, the conductivity demonstrated a pattern of initial growth, followed by a decline, and then leveling off at a constant value. Interestingly, no distinct peak conductivity was observed at a static pressure of 0.4 MPa. Therefore, we repeated conductivity tests on the sample obtain under 0.4 MPa. Result is shown in Fig. 6d, which shows a maximal conductivity of around 9,000 S/m.

Fig. 4e reveals that the average number of graphene layers remained unchanged after 40 min of ultrasonic treatment under all different static pressures. Combination with the findings from Fig. 2a, which showed a slight decrease in D50 value at 0.2 MPa, we normalized the number of graphene layers to 5.8 at 0.2 MPa to establish a relationship between conductivity and D50 value after 40 min of treatment. We extended this approach to samples obtained under other static pressure parameters, and the results are illustrated in Fig. 6e. A linear relationship was observed. Notably, these figures reveal that fewer layers result in higher electrical conductivity, with the order being 0.0 MPa < 0.2 MPa < 0.4 MPa < 0.6 MPa. It was also observed that different lateral sizes exhibit distinct conductivities, with smaller sizes resulting in lower conductivity. The variation range of the mean layer number is also essential. The slope goes steeper with higher static pressure. At 0.2 to 0.6 MPa, the slope is 889.38, 861.35, and 931.81, respectively, while it being 484.28 at 0.0 MPa since the variation of the layer number of ± 1.76 at 0.0 MPa is significantly higher than the case of the other pressures.

The electrical conductivity of graphene arises from its unique structure. The carbon atoms in graphene form a flat hexagonal lattice through sp^2^ hybridization, allowing electrons to move freely and enhancing its conductivity. Adding CMC to a graphene slurry as a binder can affect conductivity if the diameter of the graphene sheets is small. Because CMC will fill the gaps between the sheets. And the number of layers greatly affects graphene's conductivity because of the difficulty in transferring electrons between layers. To summarize, the electrical conductivity depends on the number of graphene layers and their two-dimensional dimensions. Fewer graphene layers can enhance conductivity by providing better pathways for electron migration, while an excessive number of layers or inappropriate CMC filling can obstruct these pathways and lower overall conductivity [53].

Using ethanol dissolution, PVP was removed from the graphene, resulting a pure graphene film. This film exhibited a square resistance of 1.67 Ω/m^2^ and a thickness of 1.5 μm, with an electrical conductivity of 4.0 × 10^5^S/m. Unfortunately, due to equipment limitations, we could only test films that were 2 μm thick. However, our results are consistent with those found in Ref. [54], which reported a conductivity of 3.92 × 10^5^ S/m for a film thickness of 1.875 μm. While this conductivity is lower than that of 3.71 μm thick graphene film, which was measured 1.18 × 10^6^ S/m.

## 5 Mechanism of the effect of static pressure on ultrasonic exfoliation

Boosting ultrasonic power can improve the exfoliation area. And increasing static pressure can lead to greater graphite expansion and weaker van der Waals forces [55]. Expanded graphite shown in Fig. 1c exhibits many irregular surfaces characterized by a multiplicity of furrows and fissures. When mixing expanded graphite with water, these furrows and fissures harbor air bubbles called weak regions or heterogeneous nucleation sites in the cavitation theory. This structure facilitates the formation of cavitation bubbles. It is reported that cavitation produces shock waves that expand the top section of the graphite flake, promoting interlayer relaxation [56]. The pressure from these waves measures between 1.0 MPa and 45.1 MPa, causing microjets, which break the relaxed graphite layers at speeds of up to 100 m/s. This part of the mechanism is clear enough, but the above references did not mention why these effects are present. Therefore, it is necessary to start with the behavior of bubbles under high static pressure.

Koch has demonstrated that increasing the static pressure can enhance smaller bubbles' shape and positional stability under high driving forces, as inferred through the calculation of linear resonant radii [26]:

(1) $$V_{o}=\frac{1}{2\pi R_{\mathrm{no}}\sqrt{p}}\sqrt{3\gamma \left(p_{\mathrm{stat}}+\frac{2\sigma }{R_{\mathrm{no}}}-p_{v}\right)-\frac{2\sigma }{R_{\mathrm{no}}}}$$

**Fig. 7:**
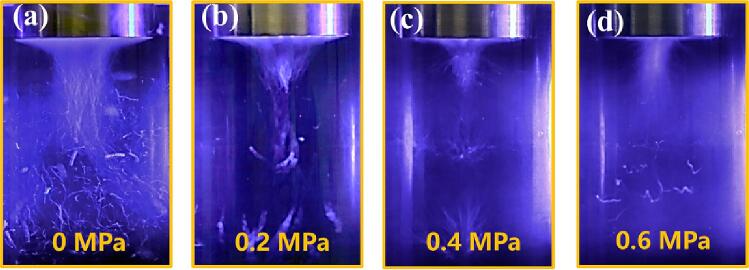
(a)-(d) The cavitation bubbles and clouds under different static pressures.

The equation has several known constants, including V_a_ at 20 kHz, assuming V_a_ equals V_o_, water's basic parameters, liquid density ρ at 1000 kg/m^3^, surface tension σ at 0.072 N/m, vapor pressure (water) P_V_ at 2.33 kPa, static pressure P_stat_ ranging from 0.0 MPa to 0.6 MPa, and adiabatic index γ at 1.67. After calculations, it was determined that the linear radius R_no_ ranges from 25.9 μm to 435.1 μm within the static pressure range of 0.0 MPa-0.6 MPa. Within a specific range, cavitation bubbles are formed and maintained in a stable location. However, if the bubbles surpass the linear resonance radius, they may fuse and eventually grow into larger bubbles that escape. This phenomenon is depicted in Fig. 7a-d. The camera captures their shape as fibrous form when they are larger and shape as more nebulous form when they are smaller. It can be clearly seen that as the static pressure increases, large bubbles decrease while small bubbles stay in a relatively fixed position. When bubbles are under high static pressure and releasing energy, only nonlinear oscillations (cavitation) can facilitate the process. Consequently, high static pressure minimizes kinetic energy loss due to the stable position of bubbles, thereby enhancing exfoliation efficiency through releasing energy in the form of cavitation collapse. The intensity of transient cavitation events increases with higher static pressure, reaching a maximum of 30 MPa [23]. Graphene sheets typically have an interlayer spacing of 0.34 nm. However, standing waves can cause the expansion of this spacing within the graphite layers. The minimum bubble size at the end of the collapse is less than 1 Å [22]. This reduction makes it easier for the bubble to occupy space within the graphite layers, ultimately disrupting the van der Waals forces. This, in turn, improves graphene exfoliation.

The exfoliation mechanism from the viewpoint of cavitation bubbles is shown above. Next, the exfoliation mechanism is explained in graphene materials. The process of graphite exfoliation can be divided into three stages [57]. In the first stage large graphite flakes are broken into fragment by ultrasound, creating wrinkled textures on their surfaces in the meantime. In the second stage cracks develops and progressively exfoliates the graphite flakes through solvent intercalation. Finally, these graphite flakes are fully exfoliated into graphene sheets. The second step is very critical. And the edge of the crack is mainly in the shape of zigzag, and oxygen free radicals exist here. This leads to a change in the chemical properties of the graphite surface and plays a crucial role in crushing the flakes. As per the findings in Ref. [58], it has been observed that graphene nanodots are generated at high power levels, resulting in an average diameter of 17 nm when subjected to 1000 W of ultrasound power. This phenomenon is attributed to forming more free radicals at high ultrasonic power, which exhibit heightened reactivity and can lead to the fragmentation of graphene nanosheets into nanodots. Both of these references mention the effect of free radicals on graphite surface fragmentation.

**Fig. 8:**
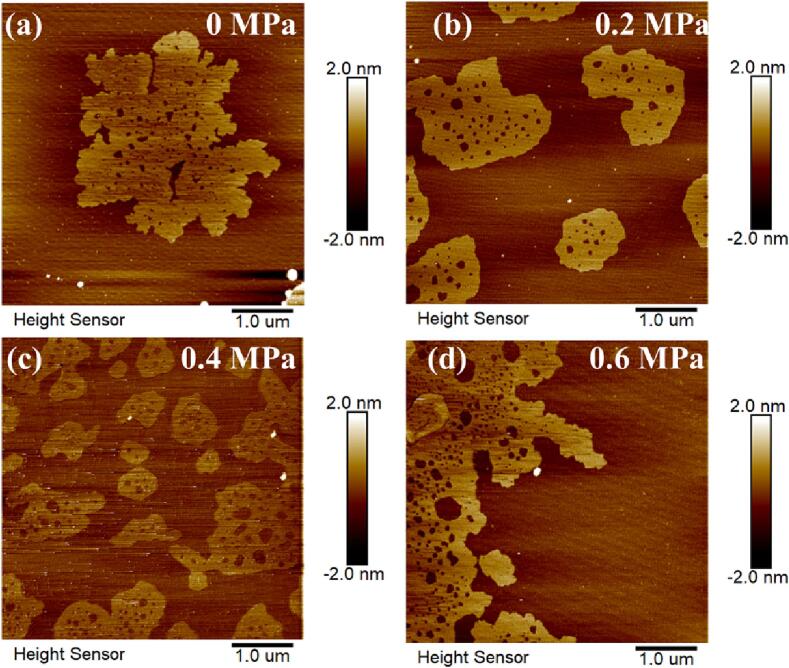
(a)-(d) The AFM image of porous single-layer graphene under different static pressures.

The most intuitive way to reflect the crushing of graphene by pressurization should be focused on the graphene nanodots. In fact, the nanodots are already present in Fig. 5. However, since the nanodots are too small to observe, we performed AFM scanning on the surface of the single-layer graphene to obtain information on the effect of pressurization on graphene. The results are shown in Fig. 8a-d. The existence of porous can be seen, with pore area ranging from 0.005 μm^2^ to 0.142 μm^2^. Interestingly, we noticed a striking 28.4 times difference in pore sizes between 0.6 MPa and 0.0 MPa. This result is in line with the high I_D_/I_G_ values previously measured in Raman spectra. This phenomenon also explains why there is almost no change in number of layers after 40 min of treatment under 0.6 MPa pressure. The graphene is already thin enough. Subsequent fragmentation creates smaller graphene nanodots. The exfoliation and fragmentation effects of graphene are enhanced at high static pressure.

Increasing static pressure can significantly impact the morphology and quality of graphene. When pressure increases, porous graphene has more edge defects and larger pore area. This effect can be advantageous for applications that require specific morphologies as it reduces π-π interaction and prevents aggregation. Applying static pressure during ultrasonication process is a third option for producing microporous graphene in addition to high-energy particle radiation and chemical treatment.

## 6 Conclusions

This study is focused on the influence of static pressure on exfoliation of graphene using a pressurized circulating ultrasonic LPE device. The proposed process is environmentally friendly and efficient. The characterization results demonstrate significant influence of static pressure. The main research findings are as following:

1. The optimal process parameters for obtaining graphene with least number of layer and largest lateral size was found to be 40-minute processing time under a static pressure of 0.6 MPa. The obtained graphene slurry without centrifugation exhibits an average lateral size of 7 μm and average number of layers of 3.5. The yield of single-layer and bilayer graphene was up to 16 % after centrifugation.
2. The electrical conductivity of obtained graphene increases with higher static pressure but decreases with prolonged exposure to ultrasonication. And eventually it stabilizes at a constant value. The obtained graphene conductivity was 4.0 × 10^5^ S/m at the optimal process parameters.
3. Through AFM scanning of obtained single layer graphene, presence of porous graphene was observed. The size of the pore on the graphene obtained under 0.6 MPa pressure increases to 28.4 times of which under 0 MPa. The method proposed in this work introduces an alternative way for porous graphene production.

## CRediT authorship contribution statement

**Hu Zong:** Investigation, Formal analysis, Conceptualization, Methodology, Software, Writing – original draft, Writing – review & editing. **Minhui Gao:** Conceptualization, Data curation, Investigation. **Aziz Ul Hassan Mohsan:** Writing – review & editing. **Yibiao Lin:** Resources, Validation. **Ying Zhou:** Conceptualization, Funding acquisition, Project administration, Supervision. **Lei Yu:** Resources, Validation. **Su Zhao:** Project administration, Supervision, Conceptualization, Funding acquisition, Writing – review & editing. **Yifei Li:** Methodology, Resources. **Jiahao Zhang:** Methodology, Resources.

## Declaration of competing interest

The authors declare that they have no known competing financial interests or personal relationships that could have appeared to influence the work reported in this paper.
